# Development of a Brazilian Portuguese adapted version of the Gap-Kalamazoo communication skills assessment form

**DOI:** 10.5116/ijme.583a.df42

**Published:** 2016-12-11

**Authors:** Anna Beatriz C. N. Amaral, Elizabeth A. Rider, Paula P. Lajolo, Luiz G. Tone, Rogerio M. C. Pinto, Marisa P. Lajolo, Aaron W. Calhoun

**Affiliations:** 1 Department of Oncology, Federal University of Uberlandia, Uberlandia, Brazil; 2 Department of Pediatrics, Harvard Medical School, Institute for Professionalism and Ethical Practice, and Division of General Pediatrics, Boston Children's Hospital, USA; 3 Department of Pediatrics, Ribeirão Preto Medical School, University of São Paulo, Brazil; 4 Department of Mathematics, Federal University of Uberlandia, Uberlandia, Brazil; 5 Department of Letters, Mackenzie Presbyterian University, São Paulo, Brazil; 6 Department of Pediatrics, University of Louisville, Louisville, USA

**Keywords:** Communication skills, medical education, cross-cultural translation, Kalamazoo consensus statement, validity and reliability

## Abstract

**Objective:**

The goal of this study was to translate, adapt and validate the items of the Gap-Kalamazoo Communication Skills Assessment Form for use in the Brazilian cultural setting.

**Methods:**

The Gap-Kalamazoo Communication Skills Assessment Form was translated into Portuguese by two independent bilingual Brazilian translators and was reconciled by a third bilingual healthcare professional. The translated text was then assessed for content using a modified Delphi technique and adjusted as needed to assure content validity. A total of nine phrases in the completed tool were adjusted. The final tool was then used to assess videotaped simulations as a means of validation.  Response process was assessed using exploratory factor analysis and internal structure was assessed via Cronbach’s Alpha (internal consistency) and Intraclass Correlation (test-retest reliability and inter-rater reliability).

**Results:**

One hundred and four (104) videotaped communication skills simulations were assessed by 38 subjects (6 staff physicians, 4 faculty physicians, 8 resident physicians, 4 professional actors with experience in simulation, and 16 other allied healthcare professionals). Measures of Internal consistency (Cronbach’s alpha = 0.818) and test-retest reliability (intra-class correlation coefficient = 0.942) were high.  Exploratory factor analysis confirmed the uni-dimensionality of the instrument.

**Conclusions:**

Our results support the validity and reliability of the Brazilian Gap-Kalamazoo Communication Skills Assessment Form when used among Brazilian medical residents. The Brazilian version of Gap-Kalamazoo Communication Skills Assessment Form was found to be adequate both in the linguistic and technical aspects.  The use of this instrument in Brazilian medical education can enhance the assessment of physician-patient-team relationships on an ongoing basis.

## Introduction

Communication skills and professionalism are domains of increasing importance to medical schools in Brazil.^1^ While the past focus of medical education was largely on the competent performance of practical procedural and examination skills, this has now transformed into a more holistic approach that involves the compassionate delivery of care.^2^ Unfortunately, this migration of focus is not yet reflected in current educational guidelines.  As an example, while the 2001 Curriculum Guidelines for the Graduation Course in Medicine in Brazil^3^^,^^4^ propose a structure that incorporates active teaching and learning methods the Standards of the National Medical Residency Commission^5^ highlights technical learning topics specific for each medical specialty, and communication skills are not included.  Several recommendations and international consensus guidelines have been developed to improve this situation. These recommendations address common communicative errors such as the use of inappropriate locations for discussing sensitive issues, poor budgeting of time for difficult conversations, the inappropriate use of medical jargon, and the undervaluing of truthfulness, privacy, confidence, trust and loyalty between physicians and patients.^6^^-^^8^ At present these recommendations are only beginning to impact Brazilian medical curricula.

In order to teach communication skills, it is critical to first be able to assess existing skill.  While many assessment tools exist for this purpose, the Gap-Kalamazoo Communication Skills Assessment Form (GKCSAF) has shown particular promise, and has been extensively piloted at Harvard Medical School’s Program to Enhance Relational and Communications Skills (PERCS) and the University of Louisville’s Program for the Approach to Complex Encounters (PACE).^9^^-^^13^ The GKCSAF is an assessment tool designed to assess the gaps between self-assessment, faculty or peer assessment, and parent/family assessment during simulated difficult encounters in pediatrics. This tool contains the items of the Kalamazoo Essential Elements Communication Checklist^12^ with two additional components assessing empathy and communication of accurate information, and allows for the calculation of a “gap analysis” that yields quantitative insight regarding learner self-appraisal. The tool has shown great value when applied in communication-focused simulation-based educational environments that encourage self-insight and constructive group discussions.^10^^,^^11^^,^^13^^,^^14^

Unfortunately, no tools equivalent to the GKCSAF exist in the Portuguese language, which makes the objective assessment of these skills in the Brazilian Medical School environment difficult. To fill this void, we initiated a project to translate the GKCSAF into Portuguese and to validate its linguistic and technical aspects in the Brazilian cultural setting.  By doing so, we hoped to create an assessment tool that could be reliably used for Brazilian medical education.

## Methods

### Description of the original Kalamazoo assessment tool

The GKCSAF is based on the Kalamazoo Consensus Statement^12^^,^^15^ and was modified by Calhoun, Rider and colleagues to enhance the scope of competencies assessed.^10^^,^^11^^,^^13^ The original version was validated for simulation-based environments in the domains of content, response process and internal structure.^10^^-^^13^ The tool contains items defined by the Kalamazoo Consensus Statement group as essential to healthcare communication: building a relationship, opening the discussion, gathering information, understanding the patient’s perspective, sharing information, reaching agreement on problems and plans, and providing closure.^15^ Two additional dimensions were added (demonstrates empathy, and communicates accurate information) in order to further improve the value of the tool when used to assess sensitive and complex communication situations, such as those occurring in acute care environments.^10^

The GKCSAF has three different versions: a peer observer/faculty evaluation that is completed by external observers, a self-assessment form completed by learners, and a family/standardized patient evaluation with language at a sixth grade reading level.  All versions maintain the instrument’s original structure and content.

Initial questions use five-point Likert scales for scoring, with choices ranging from 1 (poor) to 5 (excellent).  Data are then averaged within each assessment group (faculty vs peer vs self-assessment vs family/actor) to obtain composite scores.  Scores of 3 (good) or greater are considered adequate and scores lower than 3 indicate a need for improvement.

Two forced-choice rankings were added to this modified instrument. The first asks the rater to identify the trainee’s strongest three communication competencies, and the second asks for the three communication competencies most in need of improvement.  After each forced choice question, space is provided for those doing the assessment to give reasons why their choices were made.  These additional components were added to the original GKCSAF to improve the tool’s discrimination in situations where uniformly high Likert Scale Ratings are given (also referred to as the “Halo effect”).^16^

### Process of translation and cultural adaptation

The translation and transcultural validation was performed according to the international standards of the translation of survey tools.^17^^-^^20^ The translation into Portuguese was performed by two independent bilingual Brazilian professional translators who had no medical training and no previous knowledge of the concepts contained in the tool. A third bilingual health professional reconciled the translations.  A native English speaking translator fluent in the Portuguese language, and with no involvement in the initial steps of the process, then re-translated the reconciled version back into English. This version was then submitted to the authors of the original tool for comments.  A review committee consisting of the translators, the authors of the original tool, and other experts on the topic was then convened to create the final tool. Using a modified Delphi technique, these reviewers jointly analyzed the translations, the back translation, the original version and the comments of the authors. The goal of this analysis was to assure that semantic, idiomatic and conceptual equivalence existed between the original scale and the target Portuguese version.  The process was conducted with this level of detail to assure the maintenance of content validity as the tool was translated.

### The Delphi process – a deeper explanation

The Delphi process began with the distribution of all documents prior to the initial round of analysis. Respondents in this process remained anonymous, and percent agreement between document reviewers was used as the basis for consensus.  Each round’s results were then used to iteratively reassess and modify the tool.^21^^-^^23^ Minimum consensus was defined as 80% agreement or higher between reviewers or, starting in the second round, a percentage response stability of 60% or greater over a maximum of four rounds. In total, 86 sentences were subjected to this process, including the 9 competencies of communication, the instructions for completion of the questionnaire, the title, and the Likert scales.

### Final development

The researchers and a language coordinator assessed the discrepancies between reviewers to define the final draft of each item. A group of 21 participants was selected by convenience sampling, using the same inclusion criteria as the studies of the original tool, to participate in the final modifications of the finished product. Participants included 11 physicians (2 were faculty members), 2 nurses, 1 psychologist, 1 social worker, 2 resident physicians and 4 parents of children and adolescents between 0 and 17 years of age. These individuals reviewed the tool and were asked to respond in a similar manner to those involved in the Delphi process. Results of this review were compiled and tabulated, and, in cases of more than 20% of discrepancy in each item, resulted in modification according to the suggestion offered. The instrument was then subjected again to final language review.

### Validation process

The validation process was conducted from January to July 2014. Validation assessments were performed by a separate group of 38 clinicians and professional actors. Group demographics are presented in the Results section. After signing the Free Informed Consent Form, participants were subdivided into groups according to their clinical discipline.  Each observer was briefly informed about the study, scenario, and nature of the assessment tool prior to participation.  These subjects then observed and evaluated a series of videotaped simulations consisting of resident physicians (8 total) interacting with professional actors with previous experience in clinical simulation (4 total) using the final version of the translated GKCSAF tool. Each simulation contained situations involving the navigation of difficult ethical situations, including difficult decisions regarding terminal illness, risk of death, or possible disabling sequelae of care.  After watching the videos, participants completed the tool and a brief socio-demographic questionnaire.  Data were then statistically assessed to determine the new tool’s psychometric properties. Questionnaires in which more than 20% of the items remained unanswered were excluded from the analysis.

### Data analysis

Assessment of validity proceeded according to the frameworks of Messick and is presented accordingly.^24^ Content validity was addressed using the development process described above. Response process and, in particular, the uni-dimensionality of the final instrument, was assessed by the use of exploratory factor analysis.^24^^-^^26^ Factorability of the correlation matrix was performed with the Kaiser-Meyer-Olkin (KMO) test and by Bartlett’s test of sphericity. *A priori *criteria was used to test the theory or hypothesis of unidimensionality of the scale and select only one factor for analysis. To obtain the practical and statistical significance of the findings, ideal factor loading was set at > 0.55. Internal structure was examined by assessing internal consistency using Cronbach’s Alpha and test-retest accuracy using intraclass correlation coefficient (ICC).^27^ ICC was also used to calculate inter-rater reliability for those videos evaluated by 2 faculty members and 2 peer observers.^28^ The absence of similar instruments translated into Portuguese prevented assessment of the relationship of this tool to other established benchmarks of communication skill.   All statistical tests used an acceptable alpha error rate of 0.05. SPSS Statistics v 21.0 for Windows was used to calculate all statistics.

The present study was approved by Universidade Federal de Uberlândia’s Research Ethics Committee concerning Human Subjects (CEP/UFU), MG, Brazil.

## Results

### Content validity

In the first round of responses, 44/86 (51%) items obtained consensus, leaving 42 items for the next round.  In the second round, 20/42 (48%) items reached consensus, leaving 22 for the third round, which achieved consensus on 8/22 (36%) items. Of the fourteen items in the fourth round, 4/14 (29%) reached consensus, 5/14 (36%) held constant, and 5/14 (36%) remained discordant.  The final structure of the discordant items was determined by consensus between the researchers and the linguistic expert.

During the pre-test, nine suggestions of change in the sentences were made, seven concerned adjustments to the wording. One sentence was considered to be repetitive in items E (“Compartilha informações” – shares information) and G (“Conclui o diálogo” – provides closure): “Pergunta se a família tem mais alguma dúvida” –“ Asks if family has any questions  (concerns or other issues)”. The sentence was removed from item E, which retained the other sentences and was globally assessed using a single scale. Table 1 provides examples of the translated terms until the final version.

**Table 1:** Examples of the process of translation, reconciliation of items or words of the Gap-Kalamazoo communication skills assessment form

Original	Translation T1	Translation T2	Back translation (TB)	Portuguese final version
Concerns	preocupações	preocupações	preoccupations	preocupações
Questions	dúvidas	perguntas	doubt	dúvidas
Effectively	efetivamente	eficazmente	effectively	eficazmente
Elicits	elucido	elucido	evoke	esclareço
Clarifies	esclareço	esclareço	explain	esclareço
Checks	confiro	opta por mútua compreensão	verify	confiro

### Demographic description of validation raters

Thirty-eight participants made 104 observations about the videotaped healthcare situations using the tool, with an average of 11 responders per item of the translated instrument.  Of the 38, 6 were staff physicians, 4 were faculty physicians and 8 were resident physicians.  All were active in the fields of pediatric oncology, neonatal intensive care, or pediatric intensive care.  Sixteen allied health professionals including nurses, psychologists and social workers were also included, as were 4 professional actors with previous experience in clinical simulations. Eighty six percent (86%) of the respondents were women with a median age of 34 years and range of 25-52 years. Seventy six percent (76%) reported not having received any training in communication in healthcare. Each simulated scene lasted an average of 7.5 minutes. The time for completion of the entire translated instrument ranged between 5 and 15 minutes, (average 8.5 minutes).

**Table 2:** Factor loading and commonalities of the Brazilian version of Gap-Kalamazoo Communication Skills Assessment Form

Items	Factor loading	Commonalities
A. Builds a Relationship	0.7825	0.6123
B. Opens the Discussion	0.7523	0.5659
C. Gathers Information	0.8332	0.6942
D. Understands the Patient’s and Family’s Perspective	0.8105	0.6569
E. Shares Information	0.7547	0.5696
F. Reaches Agreement	0.7878	0.6206
G. Provides Closure	0.6884	0.4739
H. Demonstrates Empathy	0.7032	0.4944
I. Communicates Accurate Information	0.5937	0.3525

### Response process

KMO index was 0.864 and Bartlett’s sphericity test was 390.695 (df = 36, p<0.01), allowing the analysis of the main components. The single factor extracted (a priori criteria) explained 56% of the total variance, confirming the unidimensionality of the scale. The factor loadings and the commonality of the nine dimensions are shown in Table 2.

**Table 3:** Intraclass correlation coefficient (ICC) for communication domains of Gap-Kalamazoo Communication Skills Assessment Form for faculty and peer observers

Communication domains	Faculty	Peer observer
Builds a relationship	0,819	0,337
Opens the conversation	0,233	0,516
Gathers Information	0,544	0,849
Understands the family perspective	0,453	0,758
Shares information	0,813	0,192
Reaches agreement	0,709	0,505
Provides closure	0,711	0,381
Demonstrates empathy	0,739	-0,778
Communicates accurate information	0,583	-0,25
Overall	0,803	0,691

### Internal structure

Internal consistency calculations resulted in a Cronbach’s alpha of 0.818 (CI: 95% 0.760-0.866). The Intraclass correlation coefficient for test-retest was 0.942 (CI: 95% 0.912-0.975).

Faculty inter-rater reliability ICC scores ranged from 0.233 to 0.819 for each domain of communication. The lowest ICC’s were noted for the elements of opens the conversation, understands the family perspective, gathers information, and communicates accurate information (0.233, 0.453, 0.544 and 0.583) while the elements with the highest ICC’s were builds a relationship and shares information (0.819 and 0.813 respectively). The overall ICC was 0.803.

Peer observer (clinician team) ICC scores ranged from -0.778 to 0.849 for each domain of communication. Among peer observers only two communication domains scored > 0.7: gathers information and understands the family perspective.  The overall peer ICC was 0.691 (Table 3).

## Discussion

Any curriculum in medicine has among its goals the encouragement of reflection and the improvement of interpersonal relationships. One possible way to facilitate this is through the use of assessment tools that promote student feedback. A significant advantage of the Gap-Kalamazoo Communication Skills Assessment Form is that it can be used by less trained facilitators and reviewed throughout the medical training process to generate longitudinal performance data.^29^^-^^30^ By translating the Gap-Kalamazoo Communication Skills Assessment Form according to international standards, we have attempted to ensure the availability of an adequate Brazilian-Portuguese version to better meet the above curricular needs.  The primary goal of the process was to preserve the semantic equivalence and content of the original version, thereby minimizing possible mistakes that could arise from inaccurate translations.^17^

Linguistic adaptations were performed during the Delphi rounds and after the pre-test stage based on suggestions made by the participants. The modified Delphi technique confers greater reliability and authenticity to the translation process since the reviewers independently review the items.  By using a consensus process, we hoped to free the translation from external influences and individual bias as much as is possible. By calculating means and standard deviation item by item during each stage of the translation process, we sought to dynamically assess and quantify the thoughts and opinions of all reviewers.^19^^,^^20^ An example of this concerns item B (Table 1), which was the most controversial.  This item contained the sentence “Explains and/or negotiates an agenda for the visit” and was initially translated into Portuguese as “necessidade de combinar um novo horário de consulta”. After consultation of notes from the author, the item was entirely modified during the consensus using the Delphi technique. For a further example, item E also required alteration as it contained a sentence similar to the one in item G and this repetition was considered unnecessary. The participants and the linguistic expert decided to maintain the sentence only in item G, which did not affect the understanding of item E. The need for these adaptations illustrates that literal translations may produce meaningless assertions or repetitions in other languages, reinforcing the need for an appropriate cultural and linguistic conversion process .^31^

The cultural adaptations performed during the pre-test stage concerned only nine words, and few changes were made in the wording.  We speculate that the ease of adjustment relates to the simplicity of the language used in the initial English version.  We believe that this rigorous process offers the best evidence of the content validity of this translated tool.   

Regarding response process and internal consistency, the tool’s internal consistency and test-retest reliability among faculty raters were adequate to suggest validity in these domains and were consistent with the original psychometrics performed on the English Language Version.  Also, the unidimensionality of the scale was confirmed by exploratory factor analysis, suggesting a reliable response process.^9^^,^^10^  The results of inter-rater reliability testing, however, do differ somewhat from the English language version.  While concordance between overall impressions of communication is preserved, item-specific inter-rater reliability was diminished with respect to opening the discussion among faculty.  In addition, the demonstration of empathy and accurate communication of information domains were found to have negative ICC values among peer observers. Here we would note, however, that the validation statistics calculated for the original English tool did not include peer evaluations, and hence these are not strictly comparable.  In addition, strict correlation of the intraclass correlation coefficient values with that of the English language tool is not needed, as the ICC does not measure concordance of the concepts involved (which was the province of the original Delphi process). Rather, this statistic simply quantifies whether different raters perceive the intent of the question in the same way.  Still, the negative values strongly call into question the utility of this tool as a measure of peer response.  Among faculty raters, however, our results imply an overall acceptable agreement on item content, though more refinement of language may be needed for the specific items mentioned above.

Nevertheless, the above data does provide significant evidence as to the reliability of the tool in the environment in which it was assessed, leading to the recommendation that the translated tool, as it currently stands, can be used for formative feedback among resident physicians engaging in difficult conversations. The issues with inter-rater reliability delineated above, however, as well as our lack of ability at present to measure the correlation of tool scores with other measures of communicative skill in this population, preclude its use at present as a summative “grade” of communication competency. One or more rounds of additional development, particularly regarding the items yielding poor inter-rater reliability, are thus needed, followed by assessment of the psychometric properties of the resultant modified tool.

### Limitations

Perhaps the most significant limitation is the sample size of the study, which primarily resulted from the relatively lower number of individuals in the test institution with credentials mirroring the group assessed in the original English study.  Additionally, this lack of similar participants drove us to use convenience sampling standards. In addition, we note that the primary subject of the validation videotapes were resident physicians and not medical students.  While we postulate a sufficient degree of similarity between the groups to allow for the tool’s formative use, this may have some impact on the tool’s validity in the undergraduate medical population.   Future research on this tool using larger sample sizes including medical students is recommended.

## Conclusions

We describe the translation, cultural adaptation, and validation of an accepted English-based tool, the Gap-Kalamazoo Communication Skills Assessment Form, for the assessment of communication skills in healthcare interactions in the Brazilian Portuguese language. The validation process revealed some issues that will require ongoing iterative improvement, but strongly supports the introduction of this tool within Brazilian medical education. 

Given the currently formative nature of this tool, we suggest that medical educators first focus on the development of specific pilot modules designed to instruct learners in communication skills.   The use of simulation and standardized patients could be of benefit here, as they have been shown in multiple studies to enable the portrayal of realistic clinical situations.^9^^-^^13^ Once instructional modules such as this have been crafted, the Brazilian Portuguese Version of the Gap-Kalamazoo Communication Skills Assessment Form could then be integrated with the modules as a means of assessment and generation of verbal and written feedback.  In addition, the archived data from such programs could be used to perform additional psychometric calculations regarding the tool, thereby allowing for ongoing validation in the primary environment of use.  It is our hope that a process such as this could serve as the nidus for a more widespread penetration of communication curricula within the Brazilian medical education community.

### Acknowledgments

We would like to thank our colleagues from Federal University of Uberlandia who agreed to complete the tool and issued key opinions for the elaboration of this instrument. Their identities were preserved during the research, according to the ethical standards of the institution

### Conflict of Interest

The authors declare that they have no conflict of interest.
